# An outbreak of endogenous fungal endophthalmitis in immunocompetent individuals caused by presumed intravenous infusion contamination

**DOI:** 10.3389/fcimb.2026.1718140

**Published:** 2026-04-24

**Authors:** Xiaohan Zhang, Yulong Huang, Chishan Kang, Jinfeng Zhang, Wen Zhong, Jinping Xue, Xuesong Lin, Wenjie Wu

**Affiliations:** 1 Shengli Clinical Medical College of Fujian Medical University, Department of Ophthalmology, Fuzhou University Affiliated Provincial Hospital, Fuzhou, China; 2 Ningde Clinical Medical College of Fujian Medical University, Department of Ophthalmology, Ningde Municipal Hospital, Ningde, China; 3 Department of Laboratory Medicine, Ningde Municipal Hospital, Ningde, China; 4 Ningde Centers for Disease Control and Prevention, Ningde, China

**Keywords:** *Candida albicans*, contaminated intravenous infusion, endogenous fungal endophthalmitis, immunocompetent individuals, outbreak

## Abstract

**Background:**

An outbreak of endogenous fungal endophthalmitis (EFE) caused by contaminated intravenous infusion was identified in immunocompetent individuals. We aimed to describe its clinical characteristics and outcomes.

**Methods:**

This retrospective case series included all patients referred with EFE and had a history of intravenous infusions at the same rural clinic, between May 1st, 2024 and November 30th, 2024, to Ophthalmology Department of Ningde Municipal Hospital. Demographic and clinical data were collected. Whole-genome sequencing (WGS) and SNP-based phylogenetic analysis were performed on 7 culture-positive Candida albicans vitreous isolates.

**Results:**

The inclusion criteria were met in 26 eyes of 17 patients. All were healthy and immunocompetent. On average, patients presented after 24.3 days of symptoms. Presenting best corrected visual acuity (BCVA) ranged from 20/25 to no light perception (NLP). All patients were initially treated with pars plana vitrectomy (PPV) and intravitreal voriconazole injection followed by systemic voriconazole therapy. Vitreous cultures obtained during PPV were positive in 19 eyes, all showing growth of Candida albicans. Three months after treatment, patients’ BCVA improved significantly from a mean of 20/100 to 20/50 (p = 0.00011). All sequenced isolates clustered tightly in SNP-based phylogenetic analysis, supporting a clonal outbreak. Two patients with a final BCVA of NLP were initially misdiagnosed with noninfectious uveitis and treated with an intravitreal steroid injection at other hospitals. Since the closure of the rural clinic, no new cases have been reported.

**Conclusions:**

Primary PPV followed by systemic and intravitreal antifungal therapy and an epidemiological investigation could be effective in finding the infectious source of an EFE outbreak and achieving favorable visual outcomes. Misuse of intravitreal steroids due to incorrect diagnosis could lead to severe vision loss in individuals with EFE.

## Introduction

Endogenous fungal endophthalmitis (EFE) is a severe, vision-threatening intraocular infection caused by fungi, typically resulting from hematogenous spread into the eye (Burton et al., 2024). The primary focus of infection is frequently identified in the urinary tract, pulmonary system, or gastrointestinal tract, from where the fungal pathogens enter the bloodstream, causing candidemia or fungemia, and subsequently cross the blood-retinal barrier (Chen et al., 2022; Burton et al., 2024). Risk factors for EFE include immunocompromised states, such as diabetes, cancer, organ transplantation, chemotherapy, and HIV infection (Das et al., 2022). The treatment of EFE usually involves a combination of systemic and intravitreal antifungal therapy, along with surgical interventions (Chen et al., 2022). Early recognition and prompt intervention are critical to improve visual outcomes.

Although EFE is primarily observed in immunocompromised individuals, recent reports have indicated that it can also occur in otherwise immunocompetent individuals, typically following invasive procedures. Specifically, EFE has been documented after surgeries such as breast implant surgery (Querques et al., 2016), gastroscopy (Zhang and Wang, 2005), abortion, and dental extractions (Shen and Xu, 2009). Additionally, intravenous drug use (IVDU) has been implicated as a significant risk factor for EFE in immunocompetent individuals, with cases reported following heroin or other illicit drug injections (Modjtahedi et al., 2017; Tirpack et al., 2017).

Another emerging, though less explored, risk factor is the administration of intravenous infusions in rural settings (Gupta et al., 2000; Zhang and Wang, 2005; Chakrabarti et al., 2008; Sachdev et al., 2010; Dogra et al., 2018; Karkhur et al., 2020). In these settings, patients typically receive intravenous fluids or medications in clinics with insufficient aseptic practices, which may facilitate fungal contamination. However, existing studies are either case reports (Sachdev et al., 2010; Dogra et al., 2018; Karkhur et al., 2020) or retrospective case series (Gupta et al., 2000; Zhang and Wang, 2005; Chakrabarti et al., 2008), with infections occurring sporadically over extended periods.

The present study describes the clinical characteristics and treatment outcomes of an outbreak of EFE involving 17 immunocompetent patients who developed EFE after receiving presumed intravenous infusions in a rural clinic. Unlike earlier studies, this outbreak represents a point-source infection with a notably higher incidence over a short time frame. Given the rarity of EFE and its diagnostic challenges, this outbreak provides a unique opportunity to examine effective management strategies in real-world conditions.

## Methods

### Study design

This retrospective case series was conducted in accordance with the principles of the Declaration of Helsinki and received approval from the Ethics Committee of Ningde Municipal Hospital of Ningde Normal University (Approval No. NSYKYLL-2025-36). Given the retrospective nature of the study, the requirement for informed consent was waived by the ethics committee.

### Patients

A medical records review of all patients referred with endogenous endophthalmitis between May 1st, 2024 and November 30th, 2024, to Ophthalmology Department of Ningde Municipal Hospital was conducted beginning on March 1st, 2025. Inclusion criteria included culture-proven or clinical evidence of endogenous endophthalmitis in either eye and a history of intravenous infusions at the same rural clinic. Patients with possible exogenous causes of endophthalmitis (such as surgery or trauma), and other possible endogenous source of infection before symptom onset were excluded.

### Data collection

Demographic data included age, sex, comorbidities, and the time intervals from intravenous infusion to the onset of ocular symptoms, from symptom onset to presentation, and from presentation to pars plana vitrectomy (PPV). Clinical characteristics collected included past ocular history, presenting ocular and systemic symptoms, best-corrected visual acuity (BCVA) at presentation (reported in Snellen and equivalent LogMAR values), intraocular pressure (IOP), slit-lamp findings, fundus findings, treatment regimens, culture results, and antifungal susceptibility testing results. Systemic evaluation data, including blood tests, cardiac ultrasound, abdominal ultrasound, and chest CT, were also collected. The primary outcome measure was the last recorded visual acuity outcome at 3-month follow-up.

### Whole-genome sequencing and phylogenetic analysis

Genomic DNA from a representative sample of 7 culture-positive vitreous isolates of Candida albicans (designated CalB1 to CalB7) was extracted and subjected to whole-genome sequencing (WGS) by the local Center for Disease Control and Prevention (CDC). To determine the genetic homology of these isolates, a phylogenetic tree based on whole-genome single nucleotide polymorphisms (SNPs) was constructed, comparing the 7 sampled outbreak strains with 38 representative C. albicans reference genomes obtained from the NCBI public database.

### Statistical analysis

Statistical analysis was performed using SPSS version 27.0. Visual acuity data were analyzed after converting documented Snellen acuity to the equivalent logMAR value. Light perception (LP) and no light perception (NLP) were excluded and described separately. Vision data were checked for normality with the D’Agostino-Pearson omnibus normality test and were found to be distributed in a non-Gaussian fashion. Wilcoxon signed-rank test was used to analyze changes in visual acuity before and after treatment, while Mann-Whitney U test was used to compare post-treatment visual acuity between subgroups. A p value of < 0.05 was considered statistically significant.

## Results

### Patient demographics and clinical history

During a seven-month period in 2024, a total of 17 patients (26 eyes) were referred to Ophthalmology Department of Ningde Municipal Hospital and diagnosed with EFE. The average age of patients at the time of presentation was 54.3 years (median 51 years, range 42–79 years). There were 7 women (41.2%) and 10 men (58.8%). Nine patients were bilaterally affected. All were healthy and immunocompetent. There was no history suggestive of known predisposing factors, including recent hospitalization, uncontrolled diabetes mellitus, sepsis, liver disease, renal failure, cancer, indwelling lines, systemic surgery, organ transplantation, HIV/AIDS, intravenous drug use, hyperalimentation, and immunosuppressive therapy before the onset of the ocular symptoms. Each patient had received one or several intravenous infusions at the same rural clinic before the onset of ocular symptoms for a minor ailment, including 10 cases of fatigue, 4 cases of gastroenteritis, 3 cases of fever.

### Clinical presentation and diagnostic findings

**Figure 1:**
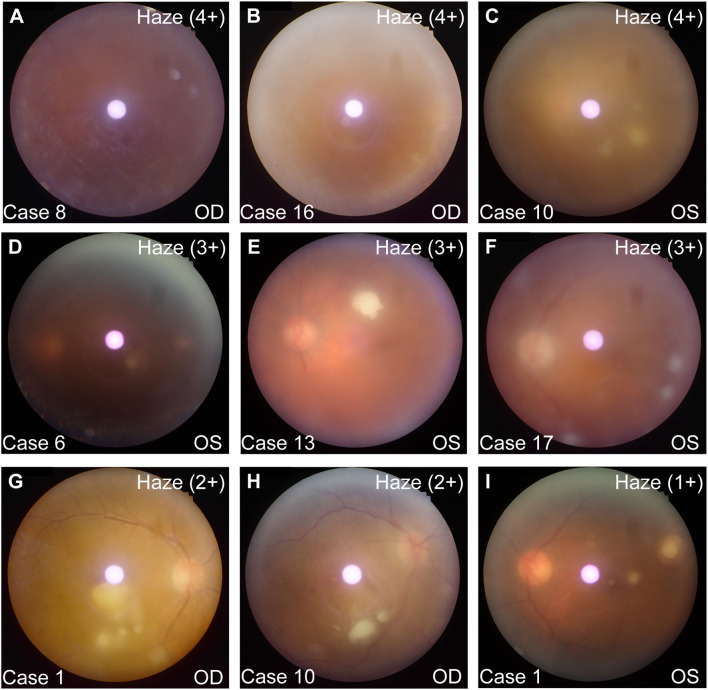
Representative fundus images and vitreous haze grading at initial presentation. **(A–C)** 4+ vitreous haze, with white snowballs visible in **(C)**. **(D–F)** 3+ vitreous haze, showing white snowballs in **(F)** and white fluffy lesions in **(D, E)**. **(G, H)** 2+ vitreous haze, featuring string of pearls in **(G)** and white fluffy lesions in **(G, H)**. **(I)** 1+ vitreous haze with white fluffy lesion.

All 17 patients fully recovered from their initial ailments within a few days, but experienced vision loss within 3 to 8 days (mean: 5.2 days) following the infusion. On average, patients presented to our department after 24.3 days of symptoms (median: 8 days, range: 2–91 days). Prior to their presentation at our institution, 5 patients had been initially managed by external ophthalmologists. Of these, one was diagnosed with cataracts and underwent cataract surgery, two were diagnosed with noninfectious uveitis and received an intravitreal steroid injection (dexamethasone intravitreal implant, 0.7 mg) along with oral corticosteroids, and two others were diagnosed with noninfectious uveitis and treated solely with oral corticosteroids. Presenting BCVA ranged from 20/25 to NLP, with a mean of 20/100 (0.6 LogMAR). Six eyes (23.1%) had a BCVA of ≥ 20/40, four eyes (15.4%) had BCVA between 20/200 and 20/40, and sixteen eyes (61.5%) had BCVA < 20/200. The mean IOP was 13.6 mmHg (range: 9–18 mmHg), measured by noncontact tonometry. The most commonly reported ocular symptoms included blurred vision, photophobia, eye pain, redness, and floaters. On initial slit-lamp examination, the following anterior segment signs were observed: conjunctival hyperemia in 12 of 26 eyes (42.3%), hypopyon in 3 of 26 eyes (11.5%), corneal edema in 5 of 26 eyes (19.2%), and keratic precipitates (KP) in 12 of 26 eyes (42.3%). All eyes exhibited vitritis of varying severity (**Figure 1**); vitreous opacities presented as a “string of pearls” in 11 eyes and as “white snowballs” in 6 eyes. Fundal details were obscured in 8 of 26 eyes at presentation, due to severe vitritis in 7 eyes and to a dense cataract in 1 eye. In the remaining eyes, focal creamy yellow-white fluffy retinal lesions were observed in 12 eyes, while multifocal lesions were noted in 6 eyes.

**Figure 2:**
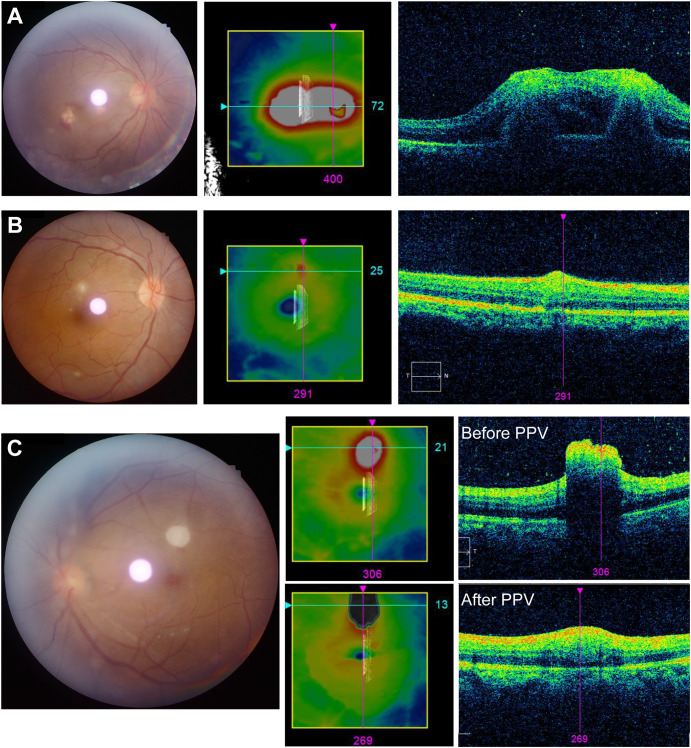
Optical coherence tomography (OCT) patterns of retinal lesions. **(A)** OCT scan showing a chorioretinal pattern of the lesion, characterized by subretinal and choroidal involvement. **(B)** OCT scan showing an intraretinal pattern of the lesion, with retinal involvement confined to the inner retinal layers. **(C)** OCT scan demonstrating the “rain cloud” sign, appearing as hyper-reflective lesion with a uniform internal pattern on OCT. Within the same affected eye, the lesion morphology evolved after PPV: the hyper-reflective “rain cloud” appearance resolved and the postoperative OCT pattern changed to an intraretinal pattern. Where available, lesion quantification was performed as supportive information. PPV, Pars plana vitrectomy.

OCT imaging was attempted in all eyes; however, due to the presence of media opacities, clear images could not be obtained in 8 eyes. Two distinct patterns of chorioretinal changes were identified on OCT, as previously described in the literature (Invernizzi et al., 2018): (1) Chorioretinal pattern, characterized by alterations in the choriocapillaris and disruption of the retinal pigment epithelium (**Figure 2A**); and (2) Intraretinal pattern, confined to the inner retinal layers and preretinal space (**Figure 2B**). In some cases, the exact lesion pattern could not be determined due to the presence of the “rain-cloud” sign. This sign is characterized on OCT by preretinal fungal aggregates appearing as round, hyper-reflective lesions with a homogeneous internal structure. These aggregates are located on the retinal surface, with the underlying retinal layers almost completely obscured by signal shadowing (**Figure 2C**). The clinical characteristics of the patients are summarized in **Table 1**.

**Table 1:** Demographic Information, Clinical Features, Microbiological Findings, and Treatment Outcomes for Each Patient.

Patient No.	Sex	Age, y	Side	Time to symptom, d	Symptom to presentation, d	Treatment before presentation	Presenting BCVA	IOP, mmHg	Hypopyon	KP	AC cell	Vitreous findings	Retinal Lesions	OCT findings	Days from symptom to PPV, d	Presentation to PPV, d	Number of Intravitreal Injections	Vitreous Culture	BCVA at 3 Months After PPV
1	M	47	R	6	6	–	CF@2ft	11	–	–	–	Vitreous haze (1+); String of pearls	1. Fovea; 2. Extramacula	1. Chorioretinal pattern; 2. Chorioretinal pattern	7	1	1	*Candida albicans*	20/500
			L	6	6	–	20/40	9	–	–	–	Vitreous haze (2+);	1. Extramacula; 2. Perifovea	1. Chorioretinal pattern; 2. Chorioretinal pattern	6	0	1	*Candida albicans*	20/32
2	M	50	R	8	15	Oral steroid	HM@2ft	14	–	+	+	Vitreous haze (4+); String of pearls^*^	1. Fovea^*^	1. Chorioretinal pattern^*^	16	1	2	*Candida albicans*	CF@2ft
			L	8	15		20/100	15	–	+	+	Vitreous haze (1+); White snowballs	1. Extramacula	1, Intraretinal pattern	15	0	2	*Candida albicans*	20/32
3	F	62	L	7	91	Oral and intravitreal steroid	NLP	10	+	+	+	Vitreous haze (4+);	Destruction of the retinal architecture with a dense accumulation of white, cotton-ball-like lesions^*^		91	0	2	*Candida albicans*	NLP
4	M	42	R	5	5	–	20/500	17	+	+	+	Vitreous haze (1+);	1. Fovea; 2. Parafovea	1. Chorioretinal pattern; 2. Chorioretinal pattern	6	1	2	Negative	20/80
			L	5	5	–	20/80	18	–	+	+	Vitreous haze (2+);	1. Parafovea	1. Chorioretinal pattern	5	0	2	Negative	20/100
5	M	47	R	7	30	–	CF@2ft	9	–	+	+	Vitreous haze (3+); String of pearls	1. Parafovea	1. Chorioretinal pattern	30	0	1	*Candida albicans*	20/25
6	M	51	R	3	2	–	20/32	11	–	–	–	Vitreous haze (1+);	1. Extramacula; 2. Parimacula	1. Chorioretinal pattern; 2. Chorioretinal pattern	2	0	2	Negative	20/32
			L	3	2	–	20/800	9	+	+	+	Vitreous haze (3+);	1. Parafovea 2. Extramacula	1. Chorioretinal pattern; 2. Chorioretinal pattern	3	1	2	*Candida albicans*	20/100
7	F	60	R	3	79	Oral and intravitreal steroid	LP	14	–	+	+	Vitreous haze (4+);	Destruction of the retinal architecture with a dense accumulation of white, cotton-ball-like lesions^*^		79	0	1	*Candida albicans*	NLP
8	M	54	R	4	8	Oral steroid	HM@2ft	15	–	+	+	Vitreous haze (4+); String of pearls^*^	1. Fovea^*^	1. Chorioretinal pattern^*^	8	0	1	*Candida albicans*	20/200
9	M	51	L	3	5	–	20/25	17	–	–	–	Vitreous haze (1+); String of pearls	1. Perifovea	1. Rain cloud sign before PPV; Intraretinal pattern after PPV	5	0	1	*Candida albicans*	20/25
10	F	53	R	4	5	–	20/80	16	–	–	–	Vitreous haze (2+); White snowballs	1. Extramacula	1. Rain cloud sign before PPV; Intraretinal pattern after PPV	6	1	1	*Candida albicans*	20/40
			L	4	5	–	CF@2ft	18	–	+	+	Vitreous haze (4+); White snowballs	1. Fovea^*^	1. Chorioretinal pattern^*^	5	0	1	*Candida albicans*	20/500
11	M	48	R	8	3	–	20/25	13	–	+	+	Vitreous haze (1+); String of pearls	1. Perifovea 2. Extramacula	1. Intraretinal pattern 2. Intraretinal pattern	4	1	1	*Candida albicans*	20/25
			L	8	3	–	20/500	15	–	–	–	Vitreous haze (2+); String of pearls	1. Fovea;	1. Chorioretinal pattern	3	0	1	*Candida albicans*	20/500
12	F	56	R	5	7	–	20/25	15	–	–	–	Vitreous haze (1+); String of pearls	1. Extramacula	1. Intraretinal pattern	7	0	1	Negative	20/20
13	F	79	R^#^	5	6	–	HM@2ft	12	–	–	–	Vitreous haze (1+); String of pearls^*^	1. Perifovea*	1. Chorioretinal pattern^*^	7	1	1	*Candida albicans*	20/63
			L	5	6	–	HM@2ft	14	–	–	–	Vitreous haze (3+); White snowball	1. Perifovea	1. Rain cloud sign before PPV; Intraretinal pattern after PPV	6	0	1	*Candida albicans*	20/100
14	F	49	R	4	31	–	20/32	14	–	–	–	Vitreous haze (2+); White snowballs	1. Extramacula	1. Intraretinal pattern;	32	1	1	Negative	20/25
			L	4	31	–	CF@2ft	15	–	–	–	Vitreous haze (4+); String of pearls^*^	1. Perifovea*	1. Chorioretinal pattern^*^	31	0	1	Negative	20/32
15	F	49	R	4	37	–	20/50	13	–	–	–	Vitreous haze (2+)	1. Perifovea	1. Chorioretinal pattern	37	0	1	*Candida albicans*	20/40
			L	4	37	–	CF@2ft	15	–	–	–	Vitreous haze (1+)	1. Fovea	1. Chorioretinal pattern	38	1	1	*Candida albicans*	20/50
16	M	55	R	5	30	–	CF@2ft	13	–	+	+	Vitreous haze (4+); String of pearls^*^	1. Fovea^*^	1. Chorioretinal pattern^*^	30	0	1	Negative	20/500
17	M	70	L	8	54	Phaco+IOL	CF@2ft	13	–	–	–	Vitreous haze (3+); White snowball	1. Perifovea	1. Intraretinal pattern	54	0	1	*Candida albicans*	20/63

^*^ Detected during intraoperative or postoperative periods;

^#^ Visualization of the fundus was obscured by a severe cataract.

BCVA, Best Corrected Visual Acuity; PPV, Pars Plana Vitrectomy; IOP, Intraocular Pressure; IOL, Intraocular len.s

All 17 patients exhibited extraocular involvement or systemic symptoms, Transient fever and cough prior to the onset of ocular symptoms were reported in 11 patients (64.7%). Blood tests were performed for all patients at presentation. Elevated white blood cell counts were detected in 35.3% of cases, while the majority showed increased levels of C-reactive protein (CRP, 64.7%), procalcitonin (PCT, 82.4%), and erythrocyte sedimentation rate (ESR, 70.6%).

### Systemic evaluation and blood culture results

All 17 patients underwent at least two fungal G-tests and blood cultures during hospitalization, all of which were negative. Notably, all 17 patients also presented with multiple pustular scalp lesions associated with hair follicles (17/17, 100%). These lesions were well-demarcated, erythematous, and in some cases, tender to touch. A series of imaging studies was performed to exclude Candida colonization in other organs. Cardiac ultrasound revealed no suspicious vegetations or intracardiac masses. Abdominal ultrasound showed no evidence of fungal infection foci. Pulmonary CT scans most commonly identified solid lung nodules (35.3%), while inflammatory changes were observed in three patients (17.6%). These three patients, diagnosed with pneumonia, subsequently underwent bronchoscopy with bronchoalveolar lavage fluid collection for culture and fungal G-test analysis. All results were negative, effectively ruling out fungal pneumonia.

### Surgical management and microbiological findings

All patients initially underwent 23-gauge pars plana vitrectomy (PPV) under local anesthesia. In cases of bilateral involvement, the second eye was operated on one day after the first. Vitreous biopsy and intravitreal voriconazole injection (0.1 mL, 50 µg/0.1 mL) were performed at the time of surgery. In one eye (Case 13, right eye), phacoemulsification was performed prior to vitrectomy due to a dense cataract. The mean interval from onset of ocular symptoms to PPV was 20.5 days (median: 7.5 days; range: 2–91 days). The mean time from presentation to surgical intervention was 0.3 days, with PPV performed within 24 hours in 17 of 26 eyes.

Following PPV of the first eye, systemic voriconazole therapy was initiated, consisting of a loading dose of 6 mg/kg intravenously every 12 hours for two doses, followed by a maintenance dose of 4 mg/kg intravenously every 12 hours for one week. The regimen was then switched to oral voriconazole at 200 mg every 12 hours for four weeks. During treatment, patients with inadequate infection control or progressive lesions received additional intravitreal voriconazole injections until effective infection control was achieved. None of the patients required secondary vitrectomy. In total, seven eyes (from four patients) received repeat intravitreal voriconazole injections.

**Figure 3:**
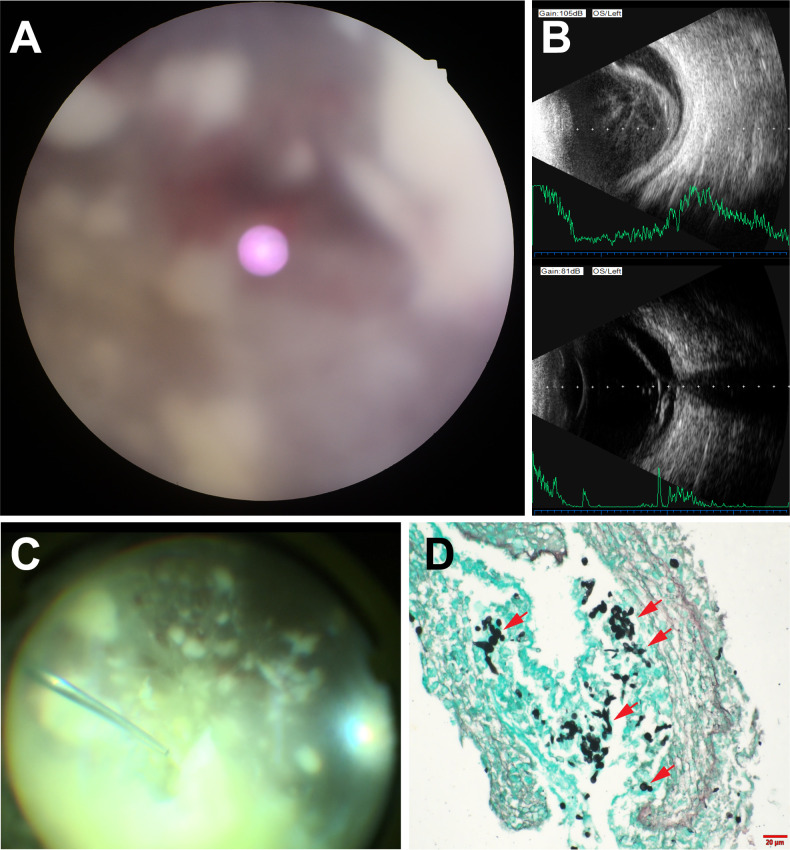
Clinical and intraoperative findings following intraocular steroid misuse (case 3). **(A)** Fundus photography showing loss of clear retinal details, with numerous cotton-ball-like white lesions and patchy hemorrhages. **(B)** B-scan ultrasonography demonstrating marked vitreous opacification and retinal detachment, consistent with extensive intraocular involvement in the setting of poor fundus visualization. **(C)** Intraoperative findings during PPV showing a dense accumulation of white, cotton-ball-like lesions on the retina, with severe destruction of the normal retinal architecture, rendering it unrecognizable, and accompanied by scattered patchy hemorrhages. A tissue sample was obtained during surgery. **(D)** Histopathological examination of the intraoperatively collected tissue sample stained with Gomori methenamine silver, revealing Candida albicans yeast forms (red arrows).

Based on intraoperative observations during PPV and postoperative OCT findings, eyes were classified into two subtypes of endophthalmitis according to lesion patterns: sixteen eyes with chorioretinal lesions (involving both the retina and choroid with vitreous infiltration) were designated as Type I, and eight eyes with intraretinal lesions (restricted to the inner retinal layers and extending into the vitreous) were designated as Type II. No eyes exhibited both chorioretinal and intraretinal patterns. Two eyes that had received intravitreal steroid injections were excluded from the classification due to loss of recognizable retinal structure (**Figure 3**). Macular involvement was present in 22 of 26 eyes (84.6%). A detailed overview of treatment protocols and intraoperative findings is provided in **Table 1**.

**Figure 4:**
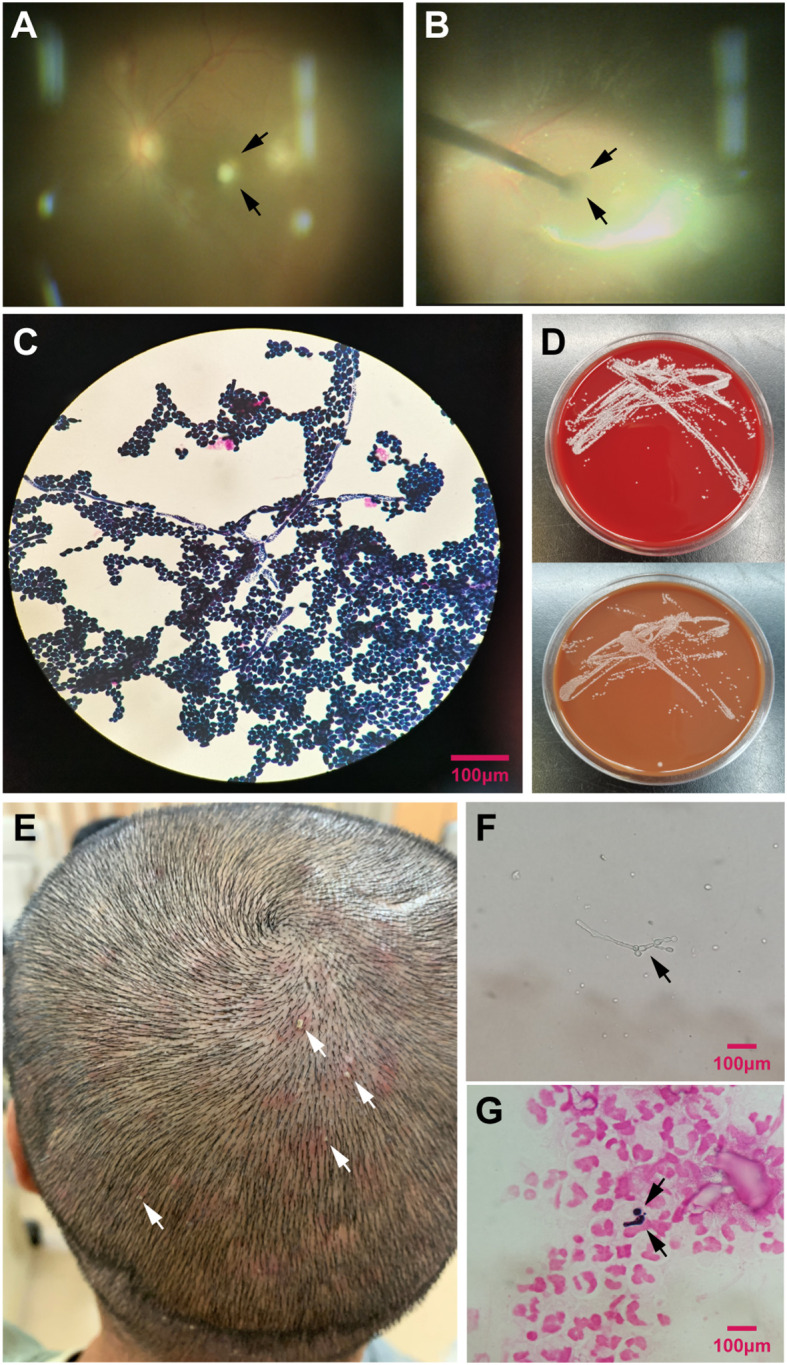
Microbiological findings in patients with endogenous fungal endophthalmitis. **(A)** Intraoperative image showing the lesion (black arrow). **(B)** Intraoperative capture of the lesion (black arrow) using internal limiting membrane (ILM) forceps. **(C)** Gram-stained microscopic examination of the specimen obtained by ILM forceps revealing numerous Candida albicans yeast forms. **(D)** Culture of vitreous fluid on Blood Agar and Chocolate Agar plates, both yielding Candida albicans colonies. **(E)** Clinical image showing multiple pustular scalp lesions in a patient. (white arrows). **(F)** Direct microscopic examination of a smear from the pustular scalp lesion fluid, demonstrating Candida albicans hyphal form (black arrow). **(G)** Gram-stained smear of the pustular scalp lesion fluid revealing Candida albicans yeast form (black arrow).

Direct microscopic examination of vitreous specimens from six eyes (23.1%) revealed fungal elements identified as Candida albicans (**Figures 4A–C**). Among the 26 eyes, vitreous cultures obtained during PPV were positive in 19 cases (73.1%), all demonstrating growth of Candida albicans (**Figure 4D**). Subsequent antifungal susceptibility testing of these positive isolates confirmed that the strains were susceptible to voriconazole. In one eye (Case 3), owing to severe destruction of intraocular structures, a small tissue sample was collected intraoperatively and submitted for pathological analysis (**Figure 3C**). Histopathological examination revealed necrotic tissue containing yeast forms of the fungus (**Figure 3D**). In four patients, samples collected from scalp pustular lesions (**Figure 4E**) were examined microscopically and consistently revealed Candida albicans hyphae and spores (**Figures 4F, G**).

**Figure 5:**
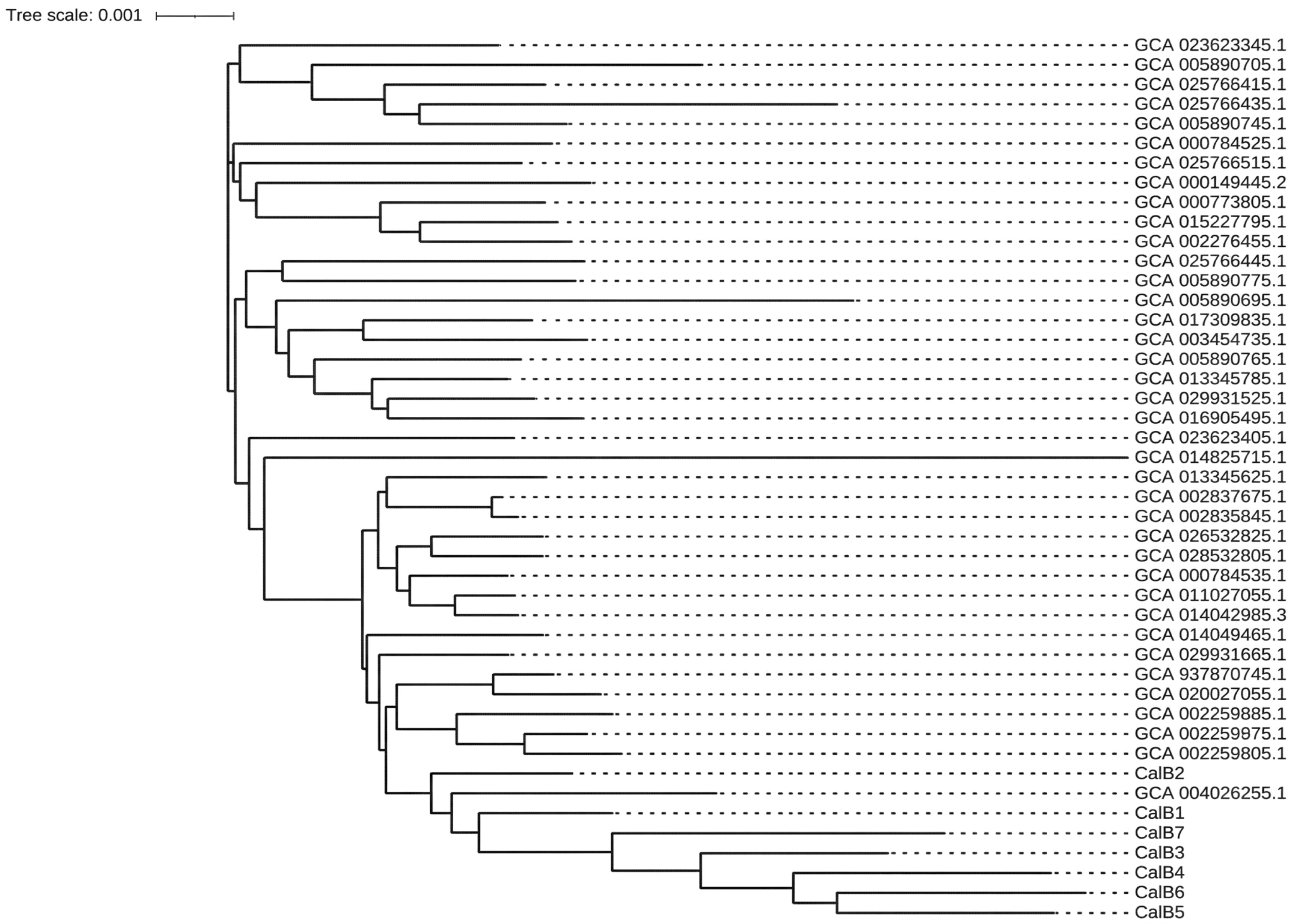
Phylogenetic tree based on whole-genome single nucleotide polymorphisms (SNPs) of Candida albicans isolates. The 7 intraocular isolates from different patients in this outbreak (labeled CalB1 to CalB7) are shown alongside 38 representative C. albicans genomes retrieved from the NCBI public database. The phylogenetic clustering demonstrates high genetic homology among the outbreak isolates, confirming a point-source clonal transmission.

To investigate the genetic relationship of the pathogen, seven viable Candida albicans isolates from the culture-positive vitreous specimens were subjected to whole-genome sequencing (WGS). The number of sequenced samples was limited to seven owing to local resource constraints and the financial coverage limitations of the epidemiological investigation at that time. Subsequent single nucleotide polymorphism (SNP)-based phylogenetic analysis revealed that all seven outbreak strains (CalB1 to CalB7) clustered tightly within the same phylogenetic branch, demonstrating high genetic homology when compared to 38 reference genomes from the NCBI database (**Figure 5**). These molecular findings strongly suggest a high probability that this cluster of infections was caused by the same clonal strain of C. albicans, which is highly consistent with a point-source exposure.

### Visual and clinical outcomes following intervention

**Figure 6:**
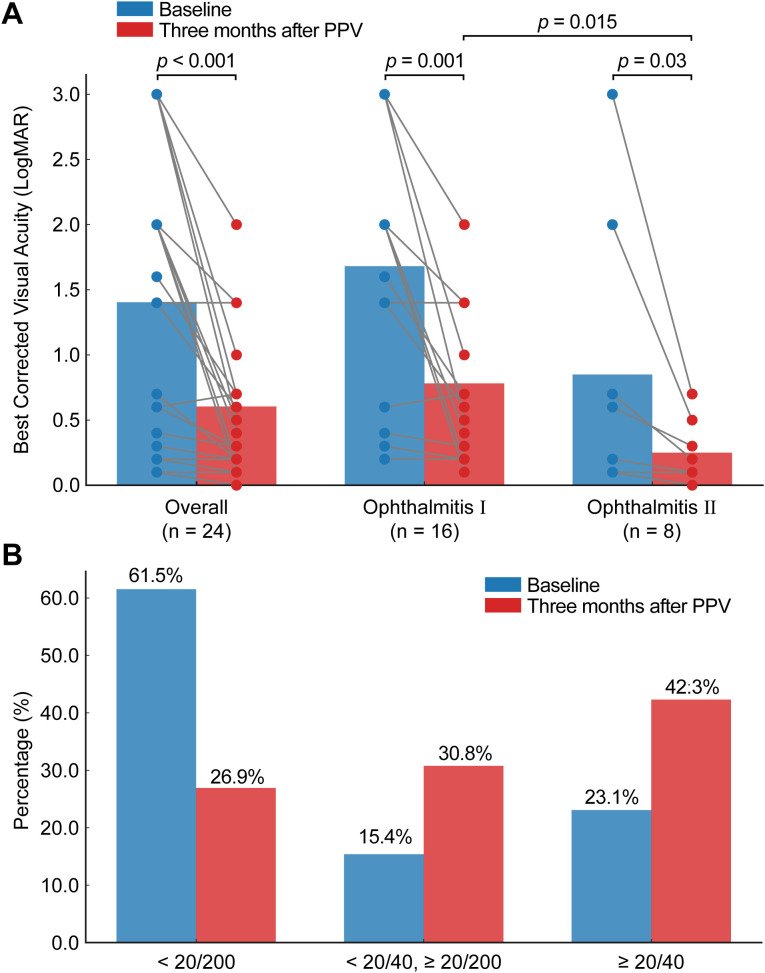
Treatment outcomes in patients with endogenous fungal endophthalmitis. **(A)** Comparison of baseline and 3-month post-surgery Best Corrected Visual Acuity (BCVA), presented for the overall population and two subgroups. Differences between baseline and post-operative BCVA were assessed using the Wilcoxon signed-rank test. Subgroup comparisons were performed using the Mann-Whitney U test. Two eyes were excluded from this analysis due to a BCVA of no light perception. **(B)** Distribution of patients across different visual acuity categories at baseline and 3 months post-surgery.

Three months after treatment, the patients’ BCVA improved significantly from a mean of 20/100 (0.60 LogMAR) to 20/50 (0.4 LogMAR) (Wilcoxon signed-rank test, W = 1.5, p = 0.00011, **Figure 6A**). At follow-up, 11 eyes (42.3%) achieved BCVA ≥ 20/40, 8 eyes (30.8%) were between 20/200 and 20/40, and 7 eyes (26.9%) had BCVA < 20/200 (**Figure 6B**). Overall, 19 eyes demonstrated improved BCVA.

Outcomes by subtype were as follows: In Ophthalmitis I, BCVA improved from 20/160 (0.9 LogMAR) to 20/63 (0.5 LogMAR) (Wilcoxon signed-rank test, W = 1.5, p = 0.001), while in Ophthalmitis II, BCVA improved from 20/50 (0.4 LogMAR) to 20/32 (0.2 LogMAR) (Wilcoxon signed-rank test, W = 0, p = 0.03). Postoperative BCVA was significantly better in the Ophthalmitis II group compared to Ophthalmitis I (mean BCVA: 20/32 [0.2 LogMAR] vs. 20/63 [0.5 LogMAR]; Mann–Whitney U test, U = 104, p = 0.015) (**Figure 6A**).

During systemic voriconazole therapy, trough serum concentrations were maintained within 1–5.5 μg/mL. One patient developed elevated liver enzymes that improved after dose adjustment and supportive hepatoprotective therapy. No other serious drug-related adverse events requiring treatment interruption or additional intervention were observed.

### Epidemiological and Molecular Investigation of the Point-Source Outbreak

In early September 2024, local and provincial health authorities, along with the CDC and the Food and Drug Administration (FDA), were notified and conducted an investigation of the rural clinic. However, the clinic had closed for unknown reasons prior to the inquiry. Although environmental sampling at the clinic was subsequently performed approximately four months after the initial infections, all cultures yielded negative results, likely due to the significant time lapse and the prior closure of the facility. Epidemiological investigations ruled out other potential sources of contamination, such as patients’ homes, workplaces, or food. Since WGS analysis confirmed that sequenced strains were highly clonal, and as all patients had received intravenous infusions at the clinic prior to symptom onset, the findings strongly implicated improper aseptic practices or contaminated fluids during infusion administration as the likely source of the EFE outbreak. The exact infusion solutions administered could not be identified, as patients were unaware of the specific substances received; thus, contamination during the manufacturing process cannot be excluded. Notably, no EFE cases related to intravenous infusions were reported from other clinics in the region. Since the closure of the clinic and up to the submission of this study, no new cases have been reported.

## Discussion

The occurrence of EFE in immunocompetent individuals is rare, and intravenous infusion in rural settings is a risk factor for its development in such individuals (Gupta et al., 2000; Zhang and Wang, 2005; Chakrabarti et al., 2008; Sachdev et al., 2010; Dogra et al., 2018; Karkhur et al., 2020). While several studies have documented the occurrence of EFE in immunocompetent patients due to intravenous infusions in rural areas, these reports are either individual case reports (Sachdev et al., 2010; Dogra et al., 2018; Karkhur et al., 2020) or retrospective case series spanning several years (Gupta et al., 2000; Zhang and Wang, 2005; Chakrabarti et al., 2008). In these studies, all cases of EFE caused by contaminated intravenous infusions were sporadic. In this study, we present the first report of a point-source outbreak of EFE in healthy, immunocompetent individuals due to presumed contaminated intravenous infusions in rural settings. As detailed in our investigation results, the epidemiological evidence strongly pointed to the rural clinic. It is worth noting that environmental sampling at the implicated clinic yielded negative results, likely because the investigation was conducted approximately four months after the initial infections, following subsequent cleaning or facility closure. However, our Whole-Genome Sequencing (WGS) analysis (**Figure 5**) successfully bridged this gap, confirming that the cultured vitreous isolates were highly clonal. Given that endogenous endophthalmitis fundamentally develops via hematogenous seeding, the combination of this definitive molecular evidence and the shared exposure history solidifies the conclusion of a point-source outbreak caused by direct intravenous inoculation of the pathogen, which also explains the concurrent transient fungemia leading to the scalp lesions observed in these patients.

In our series, all patients experienced vision loss within 3 to 8 days following intravenous infusion, which is notably faster than the typically gradual onset of EFE. For example, Chakrabarti et al (Chakrabarti et al., 2008). reported an incubation period of 10 to 25 days following presumably contaminated dextrose infusion, and Gupta et al (Gupta et al., 2000). observed a range of 7 to 86 days, with a median of 23 days. The rapid onset in our cohort may suggest a larger fungal inoculum leading to more rapid dissemination. This is consistent with Collignon et al.’s (Collignon and Sorrell, 1983) case series, where ocular symptoms appeared 3 to 7 days after injection of contaminated heroin. Moreover, 64.7% of our patients presented with flu-like symptoms, further indicating a higher fungal load or more aggressive infection compared to Tirpack et al (Tirpack et al., 2017), who found only 10% of EFE patients had systemic symptoms, and Modjtahedi et al (Modjtahedi et al., 2017), where 30% reported subjective fevers. The higher rate of bilateral involvement in our series (52.9%) is also notable, as it is more frequent than in previous studies of immunocompetent individuals, such as Tirpack et al (Tirpack et al., 2017). and Gupta et al (Gupta et al., 2000), who reported unilateral involvement in all cases. Bilateral EFE is typically seen in patients with systemic illness or sustained fungemia, as noted by Lingappan (Lingappan et al., 2012) and Tanaka (Tanaka et al., 2016), suggesting that a higher fungal load and more aggressive disease progression may result in more bilateral cases.

In our series, four patients were initially misdiagnosed with noninfectious uveitis by external ophthalmologists and treated with corticosteroids. Among them, two eyes of two patients (Cases 3 and 7), who experienced the poorest visual outcomes in this cohort, received intravitreal steroid injections at other hospitals prior to the correct diagnosis. At presentation, their BCVA was NLP and LP, respectively, both accompanied by severe destruction of the retinal structure, and the final visual acuity in both cases was NLP. Although both patients underwent fungal blood cultures and G-tests, all results were negative. The diagnosis of EFE is often challenging, and previous studies have emphasized that EFE is frequently misdiagnosed as noninfectious uveitis due to its nonspecific clinical presentation (Danielescu et al., 2020; Chen et al., 2022). In fact, rates of incorrect initial diagnosis have been reported to approach 50% (Schiedler et al., 2004) and 73% (William et al., 2017). This is particularly problematic in immunocompetent individuals, where the infectious etiology is not always considered early in the diagnostic process. Furthermore, the initial use of steroids—commonly prescribed for uveitis—can worsen the infection by suppressing the immune response, leading to disease progression (Riddell et al., 2002; Patel et al., 2014). Sallam et al. reported that patients taking systemic corticosteroids at the time when EFE developed had a higher risk of severe visual loss (Sallam et al., 2012). This underscores the critical need for heightened clinical suspicion of infectious causes in immunocompetent individuals presenting with uveitis-like symptoms, even in the absence of systemic symptoms or positive diagnostic tests. For these patients, close monitoring is essential when initiating topical or systemic steroid therapy. If there is no response to treatment, intravitreal steroid injections should ideally be avoided, or only considered after thorough exclusion of fungal causes.

The role of early vitrectomy in EFE remains a subject of ongoing debate. Some studies have suggested that early vitrectomy can improve visual outcomes and reduce the incidence of retinal detachment in EFE patients (Martínez-Vázquez et al., 1998; Sallam et al., 2012; Birnbaum and Gupta, 2016). However, other studies advocate for reserving vitrectomy for more severe cases or those refractory to medical management (Takebayashi et al., 2006; Pappas et al., 2016; Burton et al., 2024). All patients in this study were initially treated with PPV. The average time from presentation to surgical intervention was 0.3 days, with 17 of 26 eyes receiving PPV within 24 hours of presentation. We adopted aggressive treatment protocol with a low threshold for vitrectomy, serving both diagnostic and therapeutic purposes. In the context of an infectious disease outbreak, identifying the source of infection is paramount. In addition, our cases showed a high rate of macular involvement, accurate diagnosis and prompt intervention are crucial to prevent significant vision loss in these patients. Because EFE generally begins with seeding of the choroid, it has been suggested that vitreous tap may not adequately sample the vitreous cavity. Previous studies have demonstrated that vitreous samples obtained during primary vitrectomy yield a higher positive culture rate compared to those obtained from initial vitreous or anterior chamber taps (Lingappan et al., 2012; Modjtahedi et al., 2017), making vitrectomy a valuable diagnostic procedure in cases of presumed EFE (William et al., 2017). In our series, vitreous cultures obtained during PPV were positive in 19 of 26 eyes (73.1%) from 13 patients (76.5%). Epidemiological investigation revealed a shared history of intravenous infusion among these patients, and after closure of the rural clinic, no further cases were reported. Thus, the diagnostic advantage of PPV played a pivotal role in finding the infectious source. Primary PPV was also chosen for its therapeutic benefits, particularly in reducing the duration of disease progression. Vitrectomy aids in the removal of vitreous opacities, reduces the fungal load, and enhances the penetration of systemically administered antifungal agents, thus contributing to a faster resolution of infection (William et al., 2017; Bhullar et al., 2020). The decision to proceed with primary PPV was also influenced by the socio-economic constraints faced by the patient population. Most of these individuals, due to limited education and financial resources, were unable to afford extended hospital stays. Furthermore, the rural setting, coupled with significant travel barriers, made follow-up care challenging and resulted in poor compliance with outpatient visits. Given these factors, early intervention with PPV, in conjunction with intravitreal antifungal injections, was aimed at minimizing the duration of hospitalization. This approach aligns with recommendations from the recent literature, which suggest PPV in EFE cases when there is a risk of patient absconding from treatment (Wang et al., 2025).

Voriconazole, a potent broad-spectrum triazole, is characterized by its low toxicity and high ocular penetration (Riddell et al., 2011; Bihaniya et al., 2024), Recent clinical cohorts and comprehensive reviews have consistently demonstrated its robust efficacy and favorable visual outcomes in the targeted management of EFE (Bihaniya et al., 2024; Li et al., 2024). In this study, we opted not to administer systemic voriconazole prior to the first vitrectomy to optimize the yield of vitreous cultures. Immediately following the surgery, the empirical choice of voriconazole was justified by the need for prompt broad-spectrum coverage before pathogen identification. Once the vitreous culture and susceptibility testing of the initial index patient confirmed Candida albicans with sensitivity to voriconazole, it provided crucial epidemiological guidance for the immediate management of the subsequent clustered cases. Furthermore, all patients achieved complete resolution of the infection without the need for secondary vitrectomy, demonstrating that the outbreak strain of Candida albicans was clinically susceptible to this regimen. The diagnostic yield of vitreous cultures has been shown to be highly variable (Danielescu et al., 2020), with higher success rates reported when no prior antifungal treatment is administered (Patel et al., 2014).

EFE is often associated with a low blood culture positivity rate when it occurs in immunocompetent individuals (Gupta et al., 2000; Tirpack et al., 2017). In our series, all 17 patients underwent blood cultures, all of which were negative. This suggests a transient fungemia likely caused by contaminated infusions, as the intact immune system in these patients is typically able to clear the organism from the bloodstream. However, once fungal organisms seed the ocular vessels, they are more difficult to eliminate. Despite negative blood cultures, all our patients exhibited pustular scalp lesions, and cultures from four of these patients yielded positive results. These lesions are traditionally considered a hallmark of disseminated candidiasis, particularly in IVDU (Collignon and Sorrell, 1983; Martínez-Vázquez et al., 1998). In Martínez’s study, approximately half of IVDU patients with ocular symptoms presented with scalp nodules, which the authors attributed to the immunosuppressive effects of heroin, which impairs peripheral T and null lymphocyte function (Martínez-Vázquez et al., 1998). This suppression is believed to facilitate the transformation of Candida albicans into its more virulent mycelial form. However, none of our patients were IVDU, which suggests that pustular scalp lesions can also occur in EFE patients without a history of drug use. This finding underscores the importance of a comprehensive physical examination for all patients suspected of having EFE, as such lesions may serve as an important clinical clue.

Three months after treatment, a visual acuity of 20/200 or better was achieved in 19 of 26 eyes (73.1%). Previous studies have reported varying outcomes of visual acuity following vitrectomy for EFE, with the percentage of patients achieving 20/200 or better ranging from 28.5% to 83% (Gupta et al., 2000; Zhang and Wang, 2005; Takebayashi et al., 2006; Shen and Xu, 2009; Birnbaum and Gupta, 2016). The visual acuity outcomes in patients who did not undergo PPV were either better (Takebayashi et al., 2006) or worse (Zhang and Wang, 2005) than those in the vitrectomy group. However, direct comparison between our findings and those of previous studies is challenging due to differences in fungal etiology, baseline visual acuity, and timing of PPV. Therefore, it would be scientifically unrigorous to conclude which treatment protocol is superior. Nonetheless, our findings provide valuable insight into the management of EFE outbreaks. In such situations, primary PPV, followed by systemic and intravitreal antifungal treatment, combined with an epidemiological investigation, can effectively find the infectious source of an outbreak and yield favorable visual outcomes. Among the 7 eyes with a final visual acuity of less than 20/200, all had foveal involvement, and their presenting BCVA was also below 20/200. This suggests that foveal involvement and poor initial visual acuity may be predictive factors for a poor visual outcome.

Our OCT results confirmed previous findings, revealing two distinct patterns of involvement, with the chorioretinal pattern being predominant (Stephens et al., 2017; Invernizzi et al., 2018; Zhuang et al., 2020). This supports the hypothesis that Candida can seed both the choroidal and retinal vessels, with the choroid being more susceptible due to its high capillary density and blood flow. Visual acuity outcomes differed between the two patterns, with eyes showing intraretinal pattern achieving better final acuity. This may be due to the preservation of the outer retina in these cases, consistent with previous studies (Invernizzi et al., 2018).

This study has several limitations that should be considered when interpreting the findings. First, as a retrospective study, it is subject to inherent biases, including the small sample size and lack of a comparative group. While a larger cohort would be ideal for more definitive conclusions, the rarity of EFE makes it challenging to recruit a larger number of patients. Though this non-comparative nature restricts the ability to assess the relative effectiveness of different interventions, the study provides a valuable real-world example of managing an EFE outbreak.

In conclusion, this study offers valuable insights into managing an EFE outbreak in rural settings. Primary PPV followed by systemic and intravitreal antifungal therapy and an epidemiological investigation could be effective in finding the infectious source of the outbreak and achieving favorable visual outcomes. Foveal involvement and poor initial visual acuity may be predictive factors for a poor visual outcome. Misuse of intravitreal steroids due to incorrect diagnosis could lead to severe vision loss in immunocompetent individuals with EFE. These findings provide an important reference for managing similar cases in the future.

## Data Availability

The original contributions presented in the study are included in the article/supplementary material. Further inquiries can be directed to the corresponding authors.
